# Profiles of Immune Cell Infiltration in Carotid Artery Atherosclerosis Based on Gene Expression Data

**DOI:** 10.3389/fimmu.2021.599512

**Published:** 2021-03-25

**Authors:** Long Wang, Beibei Gao, Mingyue Wu, Wei Yuan, Ping Liang, Jinyu Huang

**Affiliations:** ^1^Key Laboratory of Clinical Cancer Pharmacology and Toxicology Research of Zhejiang Province, Department of Cardiology, Affiliated Hangzhou First People's Hospital, Zhejiang University School of Medicine, Hangzhou, China; ^2^Key Laboratory of Clinical Cancer Pharmacology and Toxicology Research of Zhejiang Province, Center for Translational Medicine, Affiliated Hangzhou First People's Hospital, Zhejiang University School of Medicine, Hangzhou, China; ^3^Department of Cardiology, Nanjing University Medical School, Nanjing, China; ^4^Department of Cardiology, Jiangsu Taizhou People's Hospital, Taizhou, China; ^5^Key Laboratory of Combined Multi-Organ Transplantation, Ministry of Public Health, The First Affiliated Hospital, Zhejiang University School of Medicine, Hangzhou, China; ^6^Institute of Translational Medicine, Zhejiang University, Hangzhou, China

**Keywords:** immune infiltration, immune cells, atherosclerosis, gene expression, T cells

## Abstract

Since immune infiltration is closely associated with the progression and prognosis of atherosclerosis, we aimed to describe the abundance of 24 immune cell types within atherosclerotic tissues. In the current study, we used the Immune Cell Abundance Identifier (ImmuCellAI), a web-based tool, to estimate the abundance of 24 immune cells based on the microarray profiles of atherosclerotic carotid artery samples to analyze the proportions and the dysregulation of immune cell types within carotid atherosclerosis. We found that atherosclerotic immune cells had a diverse landscape dominated by T cells and myeloid cells and that macrophages and dendritic cells (DCs) showed different abundance in normal and atherosclerotic tissues. Moreover, the expression of macrophages was closely related to the level of the expression of DCs and of exhausted T cells, while the expression of T-helper type 1 (Th1) cells was strongly correlated with the expression of T-helper type 2 (Th2) cells and effector memory cells. Our data confirm a distinct profile of atherosclerosis-infiltrating immune cell subpopulations, which may inspire an immunological direction for research on atherosclerosis.

## Introduction

The immune system, comprising various immune mediators and cells, is carefully orchestrated and is critical for the host defense (1). Innate immune cells [e.g., macrophages, neutrophils, dendritic cells (DCs), and natural killer (NK) cells] and adaptive immune cells (e.g., B and T cells) play pivotal roles in cancers and chronic inflammatory diseases (2, 3). Immune cell dysfunction, including abnormal distributions of abundance and type, has also been recognized to be associated with atherosclerosis (4). Consequently, investigation of the abundance of immune cells and of alterations in atherosclerotic (AS) tissues could provide insights into the pathogenesis, development, regression, and treatment of atherosclerosis.

Atherosclerosis is an inflammatory disease, with various stages of the disease involving immune cells (5). Subendothelial deposition of modified lipoproteins acts as a damage-associated molecular pattern to stimulate and recruit monocytes and elicit vascular inflammation. Then, monocytes are infiltrated to differentiate locally into macrophages, whose dysfunctional lipid metabolism and reduced efferocytosis led to unresolvable inflammation (6). Previous studies confirmed T cells as critical drivers and modifiers of the pathogenesis of atherosclerosis. CD4+ T-helper type 1 (Th1) cells promote the development of inflammatory atheroma while regulatory T (Tregs) cells have antiatherogenic functions (7). Little is understood regarding the role of other Th-cell subsets, such as T-helper type 2 (Th2), Th9, Th17, and Tfh cells, and the role of CD8+ T cells in atherosclerosis, which seem to have both proatherogenic and antiatherogenic functions (8). Several B lymphocyte subsets contribute to the inflammatory process through cellular and humoral responses (9). DCs are important modulators of atherosclerosis, which connect the innate and the adaptive immune responses (10). Compared with the Cardiovascular Inflammation Reduction Trial (CIRT) (11), the Canakinumab Anti-inflammatory Thrombosis Outcome Study (CANTOS) (12) resulted in fewer cardiovascular events in patients with stable atherosclerosis, which may be attributed to lessening the level of interleukin (IL)-1β, IL-6, or C-reactive protein. Therefore, it provided proof of the principle that anti-inflammatory treatments targeting special immune cells, pathways, or cytokines will have the potential for presenting atherosclerotic events in the future.

High-throughput sequencing technologies, such as microarray, RNA-sequencing, and single-cell sequencing, provide numerous gene expression profiles suitable for the study of the distribution of immune cells. Some methods, including cell-type identification by estimating relative subsets of RNA transcript (CIBERSORT) (13), Estimating the Proportions of Immune and Cancer cells (EPIC) (14), and Tumor Immune Estimation Resource (TIMER) (15), have advanced the notion of immune infiltration by enumerating immune cells from the bulk transcriptome data of tumor samples, and considering the critical role of T-cell subsets in atherosclerosis (8), we aimed to explore the proportion and alteration of immune cells in atherosclerosis. In the present study, we introduced the Immune Cell Abundance Identifier (ImmuCellAI), a web-based tool, to estimate the abundance of 24 immune cells from a gene expression data set, including 18 T-cell subsets in atherosclerosis (16).

In this study, we downloaded the microarray profiles of normal and AS tissues of the carotid artery from a gene expression omnibus (GEO) data set and applied the ImmuCellAI method to investigate the quantity of 24 immune cell types within these tissues. Furthermore, we explored the differences in immune cells and their functions in the initiation and development of atherosclerosis.

## Materials and Methods

### Data Acquisition

The data used in this study were acquired from the GEO database (https://www.ncbi.nlm.nih.gov/geo/query/acc.cgi?acc=GSE100927). The GSE100927 microarray profile was generated on the GPL17077 platform (Agilent-039494 SurePrint G3 Human GE v2 8x60K Microarray) from the samples of human peripheral arteries consisting of atherosclerotic plaques and control tissues. Atheromatous plaques were harvested and collected from patients who underwent carotid, femoral, or infrapopliteal endarterectomy, while atherosclerotic lesion-free healthy arteries were obtained from organ donors. Demographic and clinical details about this biocollection have been published in another study (17). We selected all 25 carotid tissues, including 11 normal and 14 stable atherosclerotic plaque samples, for a carotid atherosclerosis analysis.

### Evaluation of Infiltrating Immune Cells in Atherosclerosis

The ImmuCellAI was used to calculate the fractions of infiltrating immune cells with the default parameters. Recent studies using single-cell RNA-sequencing, ATAC-sequencing, and mass cytometry have shown a dominance of T cells in human carotid plaques (18–21), therefore, the focus of the study is on T cells. ImmuCellAI is an online tool that estimates the abundance of 24 immune cell types using the gene expression data based on a gene set signature method, including 18 T-cell subtypes and 6 other immune cells: B cells, NK cells, monocytes, macrophages, neutrophils, and DCs (16). The flow cytometric definition of immune cells was referenced at http://bioinfo.life.hust.edu.cn/ImmuCellAI. The infiltration score was defined as the sum of all percentages of 24 infiltrating immune cells.

### Visualization of the Filtered Data

The filtered data were used to visualize the immune cell percentage in tissues by using R version 3.6.1. The ggplot2 package was used to perform principal component analysis (PCA) with the percent values of immune cells within each sample as the input. The bar plot and the heatmap were plotted to show the proportions of 24 immune cell types. The correlation heatmap was drawn by using the corrplot package to visualize the correlations between 22 types of infiltrated immune cells. The violin plots showed that a differential expression of 24 types of immune cells was plotted by using the vioplot package.

### Statistical Analysis

The Wilcoxon test was conducted to compare the differences in cell composition between the two groups. The value of *p* < 0.05 was considered statistically significant.

## Results

**Figure 1:**
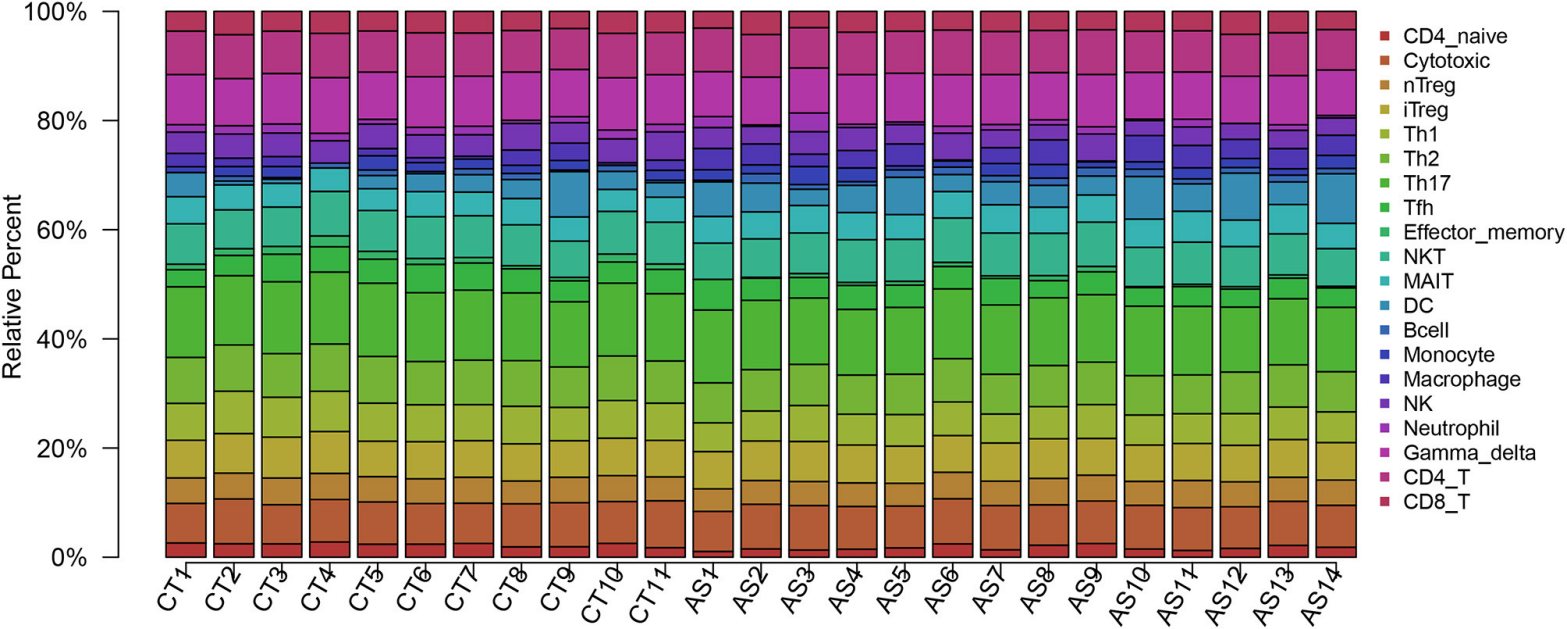
A bar plot of immune infiltration in normal samples and atherosclerosis samples. About 22 subpopulations of immune cells in 25 carotid artery samples [11 normal tissues (CT) and 14 atherosclerotic tissues (AS)] are shown.

**Figure 2:**
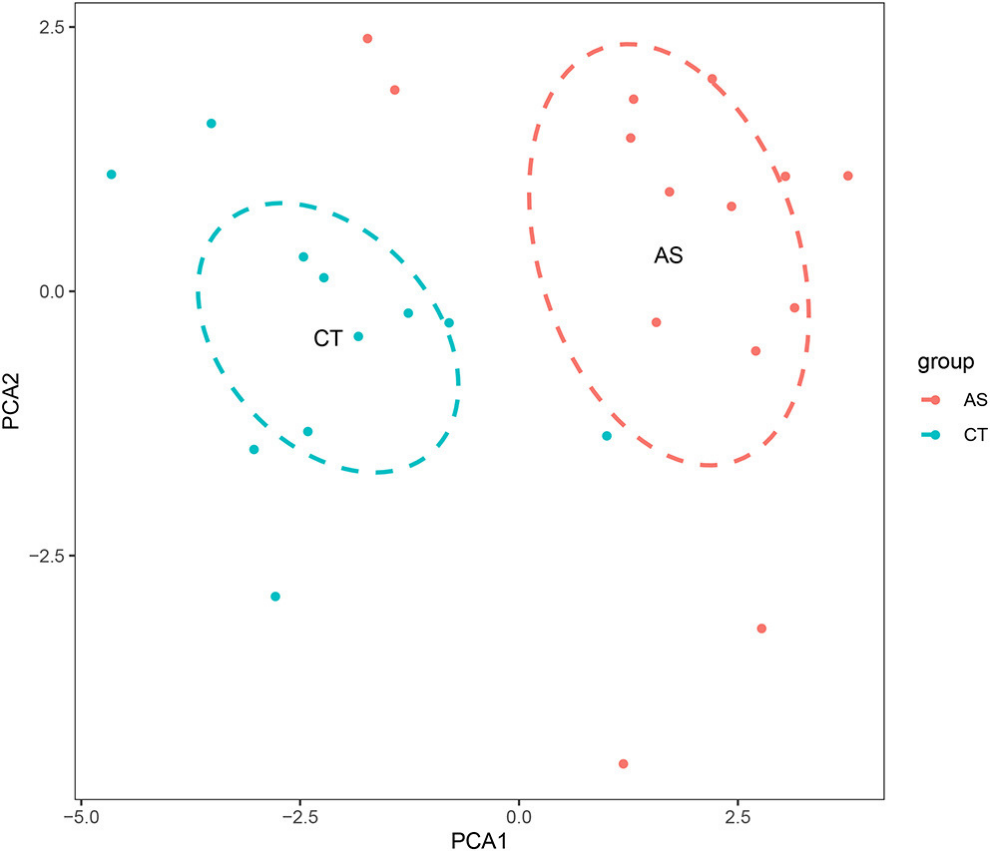
A principal component analysis (PCA) is performed on normal and atherosclerotic arteries. The first two principal components explaining the most data variation are shown.

**Figure 3:**
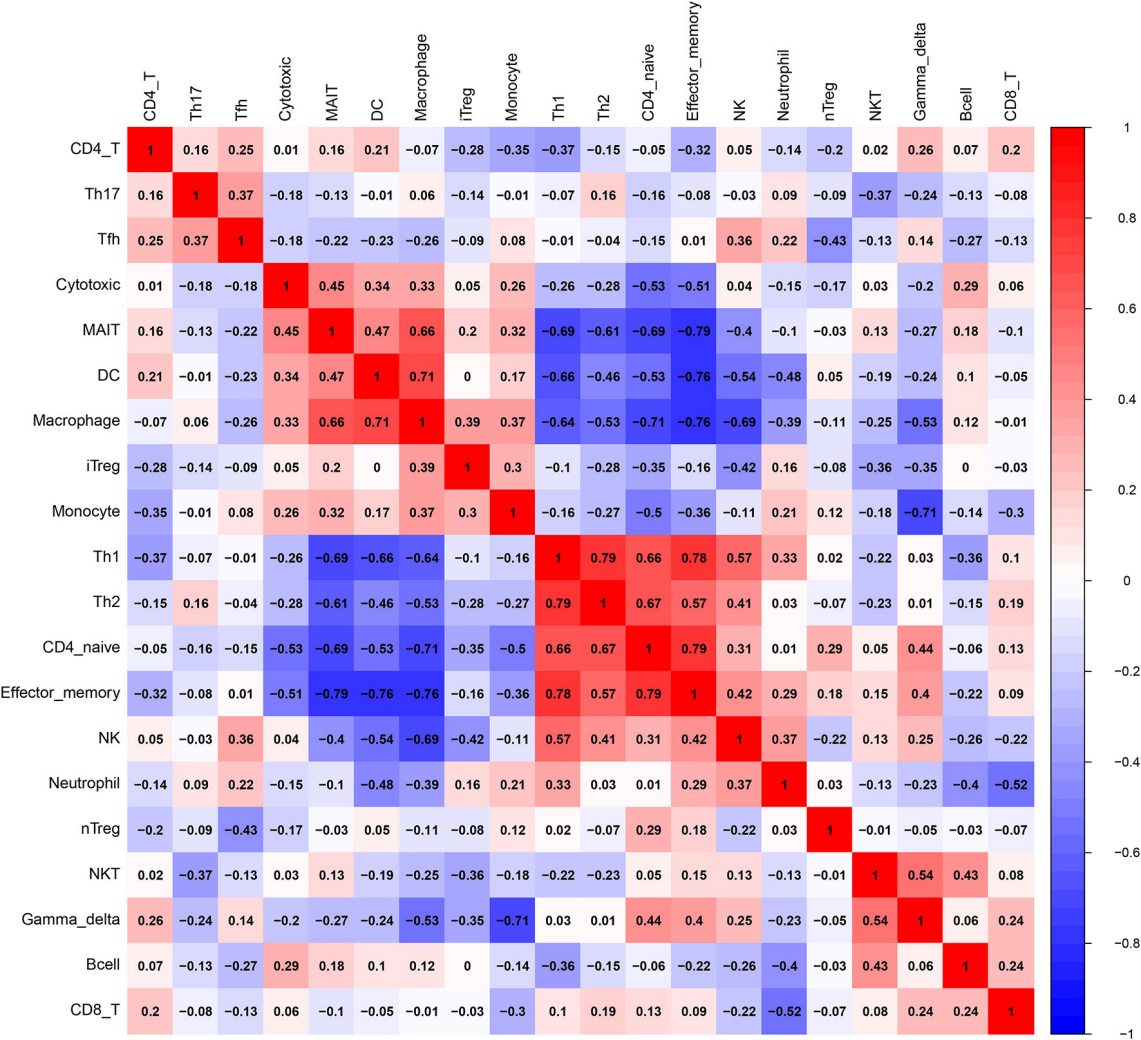
A correlation matrix of immune cell proportions between normal and atherosclerotic tissues.

**Figure 4:**
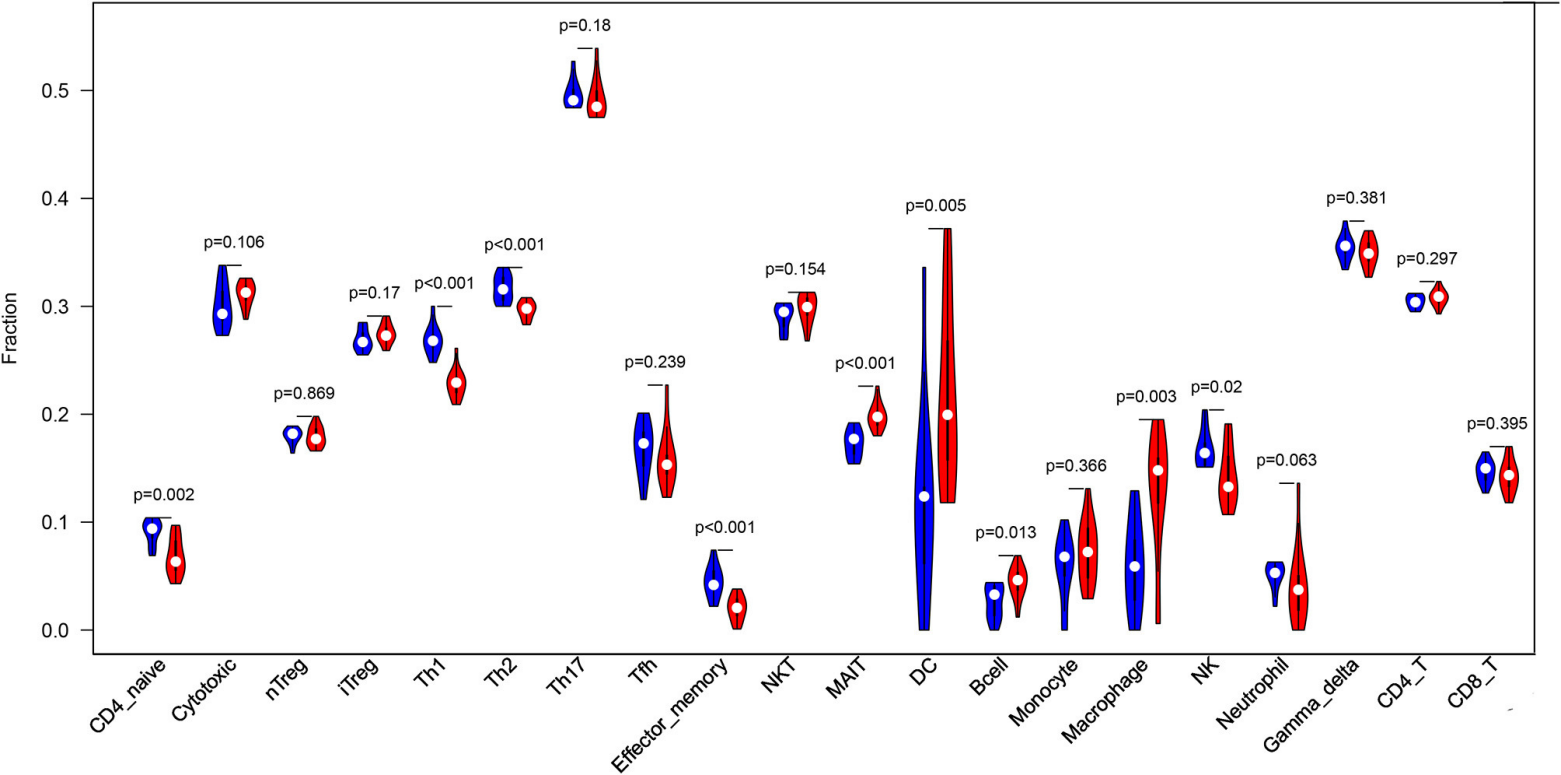
A violin plot of the immune cell proportions in the two groups. The normal control group is marked in blue, and the AS group is marked in red. The values of *p* < 0.05 were considered to be statistically significant.

**Figure 5:**
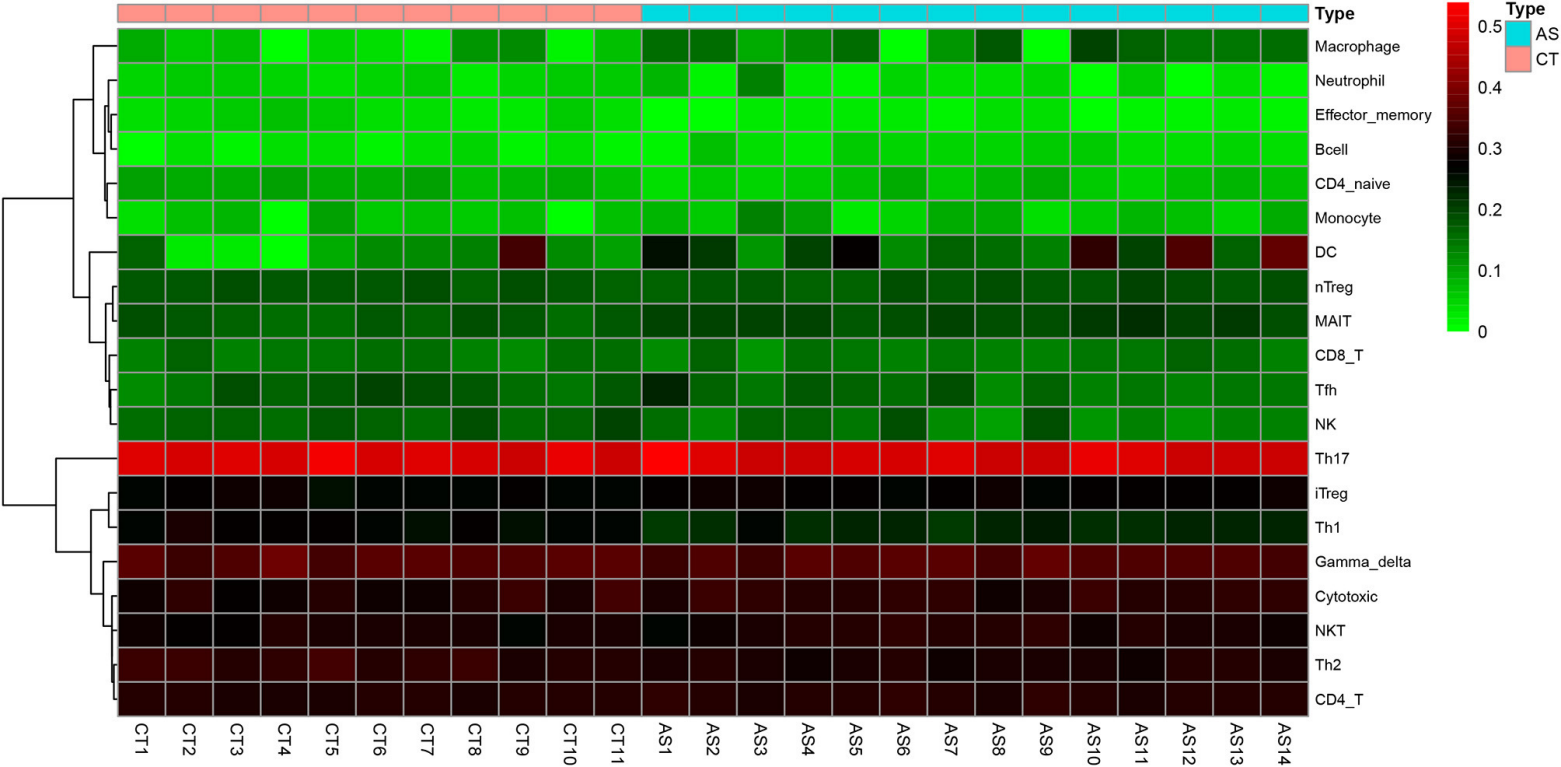
Heatmap of the proportions of 20 immune cell types in the normal group and the AS group.

Before performing an ImmuCellAI analysis, the probe names from the microarrays were replaced with the corresponding gene symbols, and the data were normalized. The ImmuCellAI method allowed a novel speculation about the immune cell infiltration in atherosclerotic carotid tissues. About 22 subpopulations of immune cells in 25 carotid artery samples (11 normal tissues and 14 atherosclerotic tissues) are shown in Figure 1 and Table 1. We found that T cells, especially Th17, γδT, Th2, and Th1 cells, dominated in the carotid artery. Because of a significant variation of immune cells in atherosclerosis, we inferred that a variation in immune cell proportions may be used to describe the difference between individuals. The abundance of immune cells from 25 samples showed a significant group-bias clustering in the PCA plot (Figure 2). The first two principal components could explain most of the data variation. In addition, we further explored the correlation between the subpopulations of immune cells and found that some subgroups had obvious correlations (Figure 3). The presence of macrophages was closely related to the expression of DCs and exhausted T cells, while Th1 cells were associated with Th2 cells and effector memory cells. Compared with normal samples, atherosclerosis samples had increased infiltration scores, which suggest a greater abundance of immune cells and altered proportions of immune cell subpopulations (Table 1, Figure 4). Atherosclerotic arteries have a higher abundance of exhausted T cells, DC cells, mucosal-associated invariant T (MAIT) cells, B cells, and macrophages. Conversely, the proportion of Th cells decreased in atherosclerosis, which could be due to the overall increase in immune cell abundance. Finally, based on a hierarchical clustering of 24 immune cell subpopulations (Figure 5), we found some modules where specific types of immune cells had a similar expression, which suggest a synergetic role in atherosclerosis.

**Table 1:** Immune cell abundance identifier (ImmuCellAI) fraction of infiltration of immune cells in atherosclerotic and normal carotid tissues.

**Immune cell type**	**Normal (%)**	**Atherosclerosis (%)**	***P*-value**
CD4_naive	4.47	3.46	0.002
Cytotoxic	5.53	6.14	0.107
nTreg	5.59	5.32	0.149
iTreg	3.77	3.43	0.166
Th1	7.26	5.34	<0.001
Th2	9.37	7.83	<0.001
Th17	13.93	12.08	<0.001
Tfh	4.57	4.48	0.501
Effector_memory	2.34	0.95	<0.001
NKT	5.23	5.19	0.767
MAIT	1.98	2.68	0.029
DC	4.25	8.14	0.002
B cell	1.23	2.17	0.001
Monocyte	2.19	2.97	0.134
Macrophage	2.39	4.94	0.003
NK	3.83	2.94	0.038
Neutrophil	2.32	1.79	0.044
γδT	9.82	9.51	0.572
CD4_T	7.42	7.12	0.222
CD8_T	2.34	2.24	0.727
Infiltration score	56.70	73.47	<0.001

## Discussion

Inherent immune diversity is a pivotal component of atherosclerosis that contributes to lesion progression or regression and predicts the clinical outcomes (20). Therefore, the identification of a specific immune dysregulation at the lesion site could provide insights into the progression of atherosclerosis. In this study, we provided a description of infiltrating immune cells in atherogenic plaques and analyzed the differences in the abundance of immune cells between the groups. These immune cells can be considered as either proatherogenic or antiatherogenic until functional experimental validation in the future.

By using the ImmuCellAI method, we can directly compare the differences in the profiles of 24 immune cells between control and atherosclerosis samples. We found that DCs, Th17 cells, γδT cells, Th2 cells, and CD4^+^ T cells were the main immune cell subpopulations in carotid artery atherosclerosis, which were consistent with the fact that T cells represent the largest population of leukocytes in atherosclerotic plaques (22). Furthermore, AS tissues have higher percentages of MAIT cells, DCs, B cells, and macrophages and lower proportions of Th1 cells, Th2 cells, Th17 cells, effector memory cells, and neutrophils. These results appear to be in contrast with the current knowledge that proatherogenic cells, including Th1 (23) and Th17 cells (24), are found at higher levels in normal tissues. The immune cell number is a relative percentage among each group. AS tissues have more immune cell infiltration, so there is a relatively lower content of Th1 and Th17 cells in these tissues than in the control tissues, which mainly contain macrophages. Due to an increased infiltration of immune cells, the decrease in percentage may not reflect a reduced number of Thlls. In case of adjusting to the infiltration score, the absolute percentage of Th cells in the AS group may not be lower than that in the normal group.

Macrophages participate in the initiation of atherosclerosis when subjected to the stimulation with oxidized lipids, cholesterol crystals, and inflammatory cytokines (25). B lymphocyte subsets are also activated and participate in the regulation of the inflammatory process (25). B1 cells have been demonstrated to inhibit lesion formation, whereas B2 cells have been shown to promote lesion formation (9). The abundance of DCs is dramatically increased during the atherosclerosis development, and DCs participate in all stages of atherosclerosis (26). DCs link innate and adaptive immune responses by presenting antigens to T lymphocytes (27) and may promote or limit atherogenesis by modulating these responses (28). CD11b+CD11c+ cells directly participate in foam cell formation (29), while conventional DCs (cDCs) interact with T cells and natural killer T (NKT) cells, resulting in an increased secretion of interferon γ (IFNγ) and IL-17 by T cells. Mice deficient in IFNγ had a lower atherosclerotic lesion burden, which suggested that IFNγ had pathogenic effects on atherosclerosis (30). The effects of Th17 cells on atherosclerosis are controversial. Some studies have shown a proatherogenic role for cytokines secreted by Th17 cells (31), while other studies have suggested that Th17 cells are associated with reduced disease development (32). Plasmacytoid DCs (pDCs) produce IFN-α and IFN-β, which play a proatherogenic role (33). Moreover, the presence of macrophages was closely related to the presence of DCs and exhausted T cells, while Th1 cells were associated with Th2 cells and effector memory cells. Th1 cells promote the progression of inflammatory atheroma, while Tregs inhibit both the innate and adaptive inflammatory responses (5). CD4+ T and CD8+ T cells were associated with increased atherosclerosis in ApoE^−/−^ mice (34). These results suggest that they may play a synergetic role in atherosclerosis. Single-cell sequencing reported that, among aortic CD45+ cells, the abundance of Th2 cells (as antiatherogenic cells) was more in the normal tissue while the abundance of macrophages was less in the normal tissue. Moreover, we used another deconvolution method (CIBERSORT) and found that the abundance of CD4 T cells is more in normal tissues than in AS tissues. However, CIBERSORT does not include Th1, Th2, or Th17 cells.

There were several limitations to our study. First, the included samples were unpaired, and we lacked the information about relevant plaque phenotypes, such as stability, vulnerability, or calcification. Second, the ImmuCellAI method focused on T-cell subsets and underestimated some macrophage or DC subsets. Finally, this was a bioinformatic analysis, and the predictions needed to be confirmed by immunofluorescent staining and flow cytometry in the future.

## Conclusion

In summary, with the help of the ImmuCellAI analysis, we revealed the immune cell composition of human carotid artery atherosclerosis and identified dysregulated immune cells. Although the study revealed some differences in respect to the literature studies, the different immunological composition of the atherosclerotic lesion suggests further investigation in the atherosclerosis studies.

## Data Availability Statement

The original contributions presented in the study are included in the article/supplementary material, further inquiries can be directed to the corresponding author/s.

## Author Contributions

WY, PL, and JH designed the study. LW performed a bioinformatic analysis. MW and BG contributed to manuscript revision. All authors approved the final submitted manuscript.

## Conflict of Interest

The authors declare that the research was conducted in the absence of any commercial or financial relationships that could be construed as a potential conflict of interest.
